# Regulation of bone mass at unloaded condition by osteocyte network

**DOI:** 10.1186/ar3568

**Published:** 2012-02-09

**Authors:** Toshihisa Komori

**Affiliations:** 1 Department of Cell Biology, Unit of Basic Medical Sciences, Nagasaki University Graduate School of Biomedical Sciences, 1-7-1 Sakamoto, Nagasaki 852-8588, Japan

## 

Disuse osteoporosis, which occurs commonly in prolonged bed rest and immobilization, is becoming a major problem in modern societies; however, the molecular mechanisms underlying unloading-driven bone loss have not been fully elucidated. Bone adjusts its shape and strength against mechanical stress. Osteocytes are the most abundant cells in bone and comprise the communication system through the processes and canaliculi throughout bone. The osteocyte network is considered to be an ideal mechanosensor and mechanotransduction system. We found that overexpression of *BCL2 *in osteoblasts reduces the number of osteocyte processes, probably due to the function of Bcl2 that modulates cytoskeletal reorganization, and induces the apoptosis of osteocytes, in which the transgene expression was reduced, presumably caused by an insufficient supply of oxygen, nutrients, and survival factors due to the reduced osteocyte processes. Our *BCL2 *transgenic mouse with accumulated dead osteocytes is a useful model to analyze the function of osteocytes, because a repair process, which replaces dead osteocytes with new osteocytes by bone resorption and formation, was not evident in the mice irrespective of the massive accumulation of dead osteocytes

We searched for the molecules responsible for disuse osteoporosis using *BCL2 *transgenic mice. Pyruvate dehydrogenase kinase isozymes (Pdk1, Pdk2, Pdk3, and Pdk4) are negative regulators of pyruvate dehydrogenase complex (PDC), which converts pyruvate to acetyl-CoA in the mitochondria, linking glycolysis to the energetic and anabolic functions of the tricarboxylic acid (TCA) cycle. Pdk4 was upregulated in femurs and tibiae of wild-type mice but not of *BCL2 *transgenic mice after tail suspension. Bone in *Pdk4*^-/- ^mice developed normally and was maintained. At unloading, however, bone mass was reduced due to enhanced osteoclastogenesis and *Rankl *expression in wild-type mice but not in *Pdk4*^-/- ^mice. Osteoclast differentiation of *Pdk4*^-/- ^bone marrow-derived monocyte/macrophage lineage cells (BMMs) in the presence of M-CSF and RANKL was suppressed, and osteoclastogenesis was impaired in the coculture of wild-type BMMs and *Pdk4*^-/- ^osteoblasts, in which *Rankl *expression and promoter activity were reduced. Further, introduction of *Pdk4 *into *Pdk4*^-/- ^BMMs and osteoblasts enhanced osteoclastogenesis and *Rankl *expression and activated *Rankl *promoter. These findings indicate that upregulation of *Pdk4 *expression in osteoblasts and bone marrow cells after unloading is, at least in part, responsible for the enhancement of osteoclastogenesis and bone resorption after unloading [1].
